# 

**Published:** 1849-02

**Authors:** 


### WESTERN JOURNAL

OF

### MEDICINE AND SURGERY:

EDITED BY

LUNSFORD P. YANDELL, M. D.,

PROFESSOR OF CHEMISTRY AND PHARMACY IN THE UNIVERSITY OF LOUISVILLE.

NO. CX.—FEBRUARY, 1849.

THIRD SERIES.

Vol. III.....No. II.

LOUISVILLE, KY:

PUBLISHED MONTHLY BY PRENTICE AND WEISSINGER,

PROPRIETORS AND PRINTERS.

1849.

